# Evergreen citrus trees exhibit distinct seasonal nitrogen remobilization patterns between mature leaves and bark

**DOI:** 10.1093/hr/uhaf103

**Published:** 2025-04-21

**Authors:** Huaye Xiong, Bin Hu, Jie Wang, Xing-Zheng Fu, Yueqiang Zhang, Xiaojun Shi, Heinz Rennenberg

**Affiliations:** Citrus Research Institute, Southwest University, Chongqing 400712, China; Center of Molecular Ecophysiology (CMEP), College of Resources and Environment, Southwest University, Chongqing 400716, China; Center of Molecular Ecophysiology (CMEP), College of Resources and Environment, Southwest University, Chongqing 400716, China; Interdisciplinary Research Center for Agriculture Green Development in Yangtze River Basin, College of Resources and Environment, Southwest University, Chongqing 400716, China; Citrus Research Institute, Southwest University, Chongqing 400712, China; Interdisciplinary Research Center for Agriculture Green Development in Yangtze River Basin, College of Resources and Environment, Southwest University, Chongqing 400716, China; Interdisciplinary Research Center for Agriculture Green Development in Yangtze River Basin, College of Resources and Environment, Southwest University, Chongqing 400716, China; Center of Molecular Ecophysiology (CMEP), College of Resources and Environment, Southwest University, Chongqing 400716, China; Chair of Tree Physiology, Institute of Forest Sciences, Albert-Ludwigs-Universität Freiburg, Georges-Köhler-Allee 53/54, 79110 Freiburg, Germany

## Abstract

Seasonal nitrogen (N) storage and remobilization are critical for tree growth. Deciduous trees primarily store N in bark; evergreen trees utilize both mature leaves and bark. Citrus is an evergreen species; leaf N storage and remobilization are well studied, but inner bark remains poorly understood. This study used pot experiments with N supply rates (low, moderate and high) to examine seasonal (winter, early, and late spring) N storage and remobilization between mature leaves (developed in autumn) and bark (main stem). Bark contains 15–35 kDa of vegetative storage proteins (VSPs), which are highly abundant and accumulate seasonally, while mature leaves contain 45–55 kDa of VSPs. Proteomic analysis revealed the oxygen-evolving enhancer protein as a key bark VSP, with Rubisco and others predominant in leaves. Under high N supply, the reduction ratio of total N content in bark from winter to early spring was higher than that in mature leaves. Under high N supply, bark arginine decreased significantly in early spring, whereas mature leaf arginine remained unchanged. Under low N supply, the decrease in proline content from winter to late spring was significantly greater in mature leaves than in bark. Thus, under high N, bark supply more arginine in early spring, whereas under low N, leaves supply more proline later. Bioinformatics indicate that ribosomal proteins may be involved in N remobilization in bark under high N and in both bark and leaves under low N. These results demonstrate that bark and mature leaves exhibit different seasonal N remobilization patterns.

## Introduction

During plant growth, nitrogen (N) remobilization is crucial for efficient utilization of N resources [1, 2], particularly by perennial woody plants [3]. This process involves the transfer of stored N from mature or senescing to new developing tissues and organs [4]. Nitrogen storage in plants primarily relies on vegetative storage proteins (VSPs) [5], which accumulate in mature tissues as a result of N remobilization from senescing leaves before abscission. These proteins typically reach high abundance during winter and serve as dynamic N reservoirs that are rapidly degraded in spring to support the development of new tissues [6]. Deciduous poplar trees typically store N in the inner bark (the living part of bark) [7] and wood with VSPs of 32–38 kDa [8–10], while evergreen trees store N in mature leaves through dual-functional proteins, which not only catalyze enzymatic reactions but also transiently act as N storage during winter [11–13], with Rubisco (ribulose-1,5-bisphosphate carboxylase/oxygenase) being a typical example [14]. Apart from mature leaves, evergreen trees can also store N in other mature tissues, including inner bark, roots, sapwood, and stems [15, 16]. The spatial diversity of storage proteins highlights the complexity of N remobilization in evergreen trees, yet inner bark- and leaf-specific mechanisms remain poorly characterized [6, 17].

To understand these mechanisms, proteomics has provided insights into the complexity and diversity of VSPs [18]. In evergreen conifers like Chinese fir and young cypress, N fertilization or winter conditions significantly enhance leaf accumulation of Rubisco and oxygen-evolving enhancer protein (OEE) [19, 20]. However, the role of OEE as a VSP is not yet conclusively established, owing to a lack of time-series analyses. In contrast, broadleaf evergreens such as citrus accumulate distinct VSPs (45–55 kDa) in mature leaves during winter, particularly under high N supply, degrading them rapidly in spring [21, 22]. This seasonal pattern indicates their role as transient N reserves for spring growth. Proteomic analyses identified Rubisco and an aspartate endopeptidase as primary VSPs in citrus tree leaves [23]. However, whether citrus trees utilize OEE as a VSP, similar to conifers, remains unclear, highlighting a critical gap in understanding N storage strategies across evergreen species.

To our knowledge, there is no definitive report demonstrating specific VSPs in citrus bark. Although several proteins in citrus bark have been initially identified under various stress conditions, their role as VSPs has not been proven [24, 25]. Furthermore, while several studies have reported variation in soluble protein concentrations between young, senescing and mature citrus leaves across different seasons, they did not identify distinct proteins present in the bark relative to those in mature leaves [21, 22]. Citrus leaves account for less than 21% of the aboveground biomass [26], with an N content ranging from 2% to 3% [27]. Although there is limited information on the biomass and N content of citrus bark, comparative data from tropical forest trees reveal that the N content in bark can reach up to 2% of dry mass [28], with bark typically accounting for about 2% to 20% of plant biomass [28]. In addition, inner bark represents approximately 17.7% of stem cross-sectional area and contributes on average 38.7% of the total stem N pool, suggesting that inner bark may be an overlooked but substantial N reservoir [7]. Additionally, the inner bark, as part of the vascular system through the secondary phloem, directly facilitates N transport and remobilization to growing tissues [7]. Therefore, it is crucial to determine whether the VSPs in bark differ from those in leaves.

In addition to VSPs, free amino acids (FAAs) are also important N turnover resources [29]. FAAs remobilized from senescing tissues accumulate in leaves and inner bark, potentially serving as N sources for VSP synthesis or directly supporting N remobilization [4].While mature citrus leaves seasonally accumulate proline and arginine to fuel spring growth, whether inner bark employs similar FAA-mediated strategies remains unknown [23]. This gap in understanding is particularly significant given that the inner bark contains the secondary phloem, directly involved in vascular transport, and contributes substantially to overall biomass, suggesting that it may serve as an important yet underexplored reservoir of FAAs.

**Figure 1:**
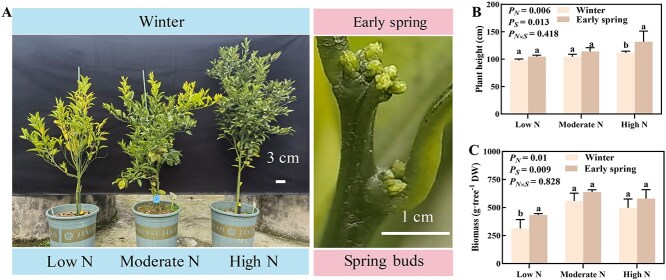
Effect of nitrogen (N) fertilizer application on citrus growth. (A) The visual growth performance of citrus trees during winter and early spring. (B and C) The height and biomass of citrus trees, respectively. Data of each treatment are expressed as means ± standard deviation (SD) of three replicates set of plants (*n* = 3). *P_N_*: nitrogen fertilizer treatment (low N, moderate N, and high N). *P_S_*: seasons (winter and early spring).

Citrus trees, being one of the most widely planted evergreen fruit trees globally, often face excessive N fertilizer application in citrus orchards, to enhance yield and fruit quality [30, 31]. From the perspective of reducing N fertilizer application and increasing system sustainability, it is important to note that the N remobilized from winter reserves in mature leaves in spring provides at least an additional 40 kg N/ha for the growth of citrus trees, indicating that natural nutrient cycling plays a crucial role in supporting citrus tree development [32]. Consequently, identifying key molecules associated with N remobilization is crucial to understand the N use efficiency of citrus plants [33–35]. Current research indicates that the synthesis of VSPs in both the bark of deciduous poplar (which sheds leaves in winter) and mature leaves of citrus trees (which retain leaves in winter) coincides with seasonal increase in the expression of ribosomal proteins and proteins involved in energy synthesis [18, 21]. Understanding of seasonal and N-driven variations in N storage within bark and leaves remains limited, despite their importance for supplying N to new growth. Clarifying the roles of these tissues in N storage and remobilization is essential for optimizing fertilization practices, improving N-use efficiency, and reducing environmental impacts.

The aim of this study was to elucidate the physiological mechanisms of N remobilization in bark and mature leaves of citrus trees. We hypothesize that (1) during spring, citrus bark and mature leaves exhibit different patterns of N remobilization; (2) the types of VSPs differ between citrus bark and mature leaves; and (3) consequently, the key molecules driving N remobilization differ between bark and mature leaves. To address these hypotheses, we conducted an integrated time-series analysis of N partitioning and proteome profiling in citrus bark and mature leaves across three distinct seasons—winter, early spring, and late spring—under varying N supply conditions (low, moderate, and high) to capture the dynamic changes in N remobilization. This comprehensive approach aims to provide a detailed understanding of the differences in N remobilization between mature leaves and bark of evergreen citrus trees.

## Results

### The growth of citrus trees is affected by N fertilization and season

For biometric analysis, we measured plant height and whole-tree biomass of citrus trees in winter and at bud break in early spring (Fig. 1A). Regardless of N fertilization, plant height (*P_S_* = 0.013) and biomass (*P_S_* = 0.009) were significantly increased in early spring (March 2022, during bud break) compared with winter (December 2021) (Fig. 1B and C). Also, N fertilization significantly enhanced plant height (*P_N_* = 0.006) and biomass (*P_N_* = 0.01) of citrus trees. Thus, the development of citrus trees is significantly affected by both N fertilization and seasons.

### Molecular weight range of storage proteins in citrus tree

**Figure 2:**
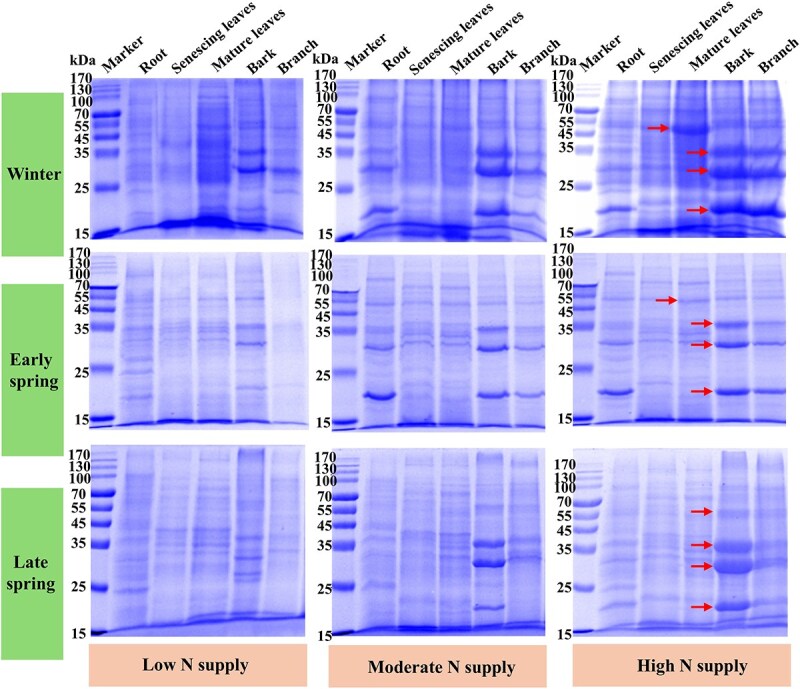
Analysis of the effect of N fertilizer application on the abundance of storage proteins (*n* = 3) in various citrus organs explored by gel electrophoresis. The arrows indicate potential VSPs (15–35 and 45–55 kDa).

We initially used sodium dodecyl sulfate-polyacrylamide gel electrophoresis (SDS-PAGE) to search for storage proteins in different tissues of citrus trees (Fig. 2). SDS-PAGE analysis demonstrated that the proteins accumulated in mature leaves during winter were primarily within the 45- to 55-kDa range. In contrast, the proteins accumulated in bark and branch during winter were primarily in the 15- to 25- and 25- to 35-kDa ranges. In early spring, the protein contents within these ranges significantly decreased compared to winter, supporting their function as storage proteins. These results indicate that mature leaves and bark accumulate different types of storage proteins. In contrast, the molecular weights of proteins accumulated in roots were similar to those detected in mature leaves, branches, and bark. Compared to low N supply, high N supply significantly increased the abundance of proteins of the 45- to 55-kDa range in mature leaves and of the 15- to 25- and 25- to 35-kDa ranges in bark.

### Seasonal effects of N application on N partitioning in bark and mature leaves

**Figure 3:**
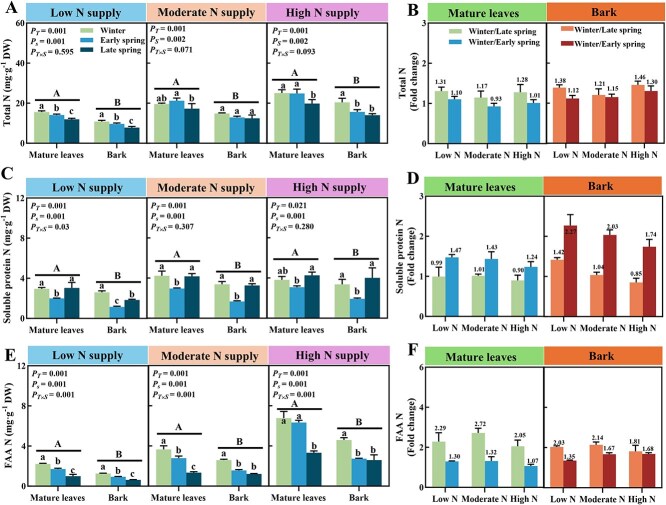
Effect of nitrogen (N) application on N partitioning in mature leaves and bark of citrus tree. (A, C, and E) Total N, soluble protein N, and FAA N (FAAN) content of mature citrus mature leaves and bark under low N, moderate N, and high N supply, respectively. (B, D, and F) The total N, soluble protein N, and FAA N contents in the bark of citrus mature trees, respectively, along with their seasonal fold changes, which are calculated as the ratios of winter to early spring and winter to late spring values. The results are presented as means ± standard deviation (*n* = 3). *P_T_*: tissues (mature leaves and bark). *P_S_*: seasons (winter, early spring and late spring). Different lowercase letters represent significant differences (*P <* 0.05) in mature leaves or bark across different seasons, while different uppercase letters indicate significant differences between bark and leaves (two-way randomized block analysis of variance, Tukey’s multiple comparisons).

At low N supply, total N contents in mature leaves and bark showed a significant decrease in early and late spring compared to winter (Fig. 3A). At high N supply, the total N content in mature leaves did not decrease in early spring but showed a significant decrease in late spring; the total N content in bark decreased significantly in both early and late spring compared to winter. Regardless of low or high N treatment, the total N content in mature leaves during winter was always higher than in bark. The fold change in total N (winter/early spring) indicated that under high N supply, the proportion of decrease in bark (fold change=1.30) in early spring was higher than in mature leaves (fold change=1.01) (Fig. 3B). However, at low N supply, the ratio of fold changes in early spring bark (fold change=1.12) and mature leaves (fold change=1.10) was not significantly different. These results indicate that at high N supply, citrus trees mobilize a larger proportion of stored N resources from the bark than from mature leaves in early spring.

Regardless of N supply, soluble protein N in both bark and mature leaves generally decreased in early spring compared to winter but increased in late spring compared with early spring (Fig. 3C). The fold changes of ‘winter/early spring’ of soluble protein N content were higher in bark (1.74–2.27) than in mature leaves (1.24–1.47) (Fig. 3D). These results indicate that remobilization of soluble protein N takes place in early spring but was no longer evident in late spring, while more of this N fraction is remobilized in bark than in mature leaves. At low and moderate N supply, the FAA N content in mature leaves and bark showed a significant decrease in early and late spring compared to winter. However, at high N supply, the FAA N content in mature leaves did not decrease in early spring as expected but showed a significant decrease in late spring; despite this observation, the FAA N content in bark still showed a downward trend in early spring (Fig. 3E). The fold change ratio of ‘winter/early spring’ for FAA N was higher in bark (1.68) than in mature leaves (1.07) at high N supply (Fig. 3F).

**Figure 4:**
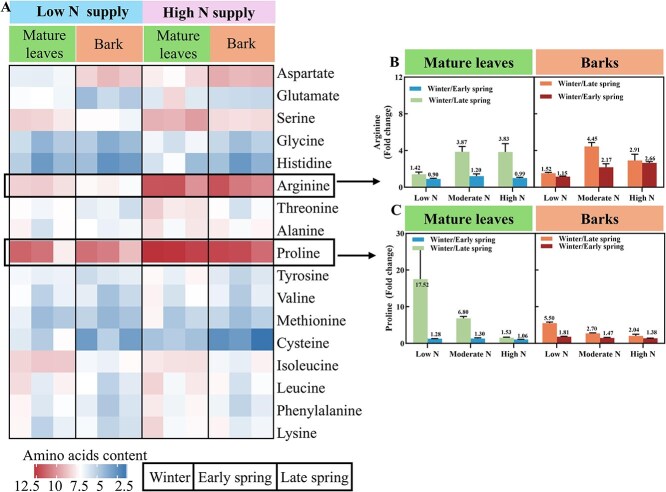
Effects of N application on amino acid composition and fold change in the bark and mature leaves. (A) The quantification of 17 FAAs. (B) Fold changes in arginine content in mature leaves and bark, respectively. (C) Fold changes in proline content in mature leaves and bark, respectively.

Among the 17 individual FAA quantified, proline was the most abundant FAA in mature leaves and bark in winter, followed by arginine, regardless of N supply (Fig. 4A). Arginine content in the bark was significantly higher during winter compared to early (fold change = 2.66) and late spring (fold change = 2.91) under high N supply (Fig. 4B). A similar decrease was observed in the mature leaves in late (fold change = 3.83) but not in early spring (fold change = 0.99). Regardless of N supply, the fold change ratios of ‘winter/late spring’ for proline was higher than the fold change ratio ‘winter/early spring’ in mature leaves and bark. At low N supply, the fold change ratios of ‘winter/late spring’ for proline were 5.50 and 17.52 in bark and mature leaves, respectively, and thus higher than at high N supply (2.04 and 1.53) (Fig. 4C). These results show that at high N supply, more arginine is mobilized in early spring from bark compared to mature leaves. Under low N supply, extensive proline remobilization from mature leaves predominantly occurs in late spring rather than in early spring.

### Identification of storage proteins in bark and mature leaves

**Figure 5:**
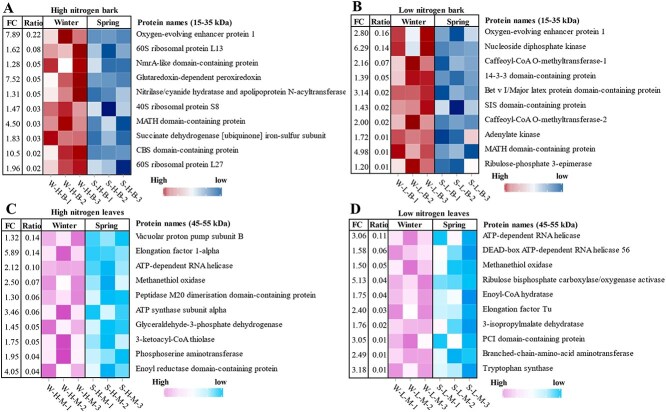
Proteome profiling of citrus leaves and bark at varying soil nitrogen (N) contents. (A and B) Proteins (*P* < 0.05, 15–35 kDa) with the highest abundance in the bark during winter and were decreased in spring under high N and low N conditions, respectively. (C and D) Proteins (*P* < 0.05, 45–55 kDa) with the highest abundance in mature leaves during winter and were decreased in spring under high N and low N conditions, respectively. S-H-B: spring-high nitrogen-bark, S-H-M: spring-high nitrogen-mature leaves, S-L-B: spring-low nitrogen-bark, S-L-M: spring-low nitrogen-mature leaves, W-H-B: winter-high nitrogen-bark, W-H-M: winter-high nitrogen-mature leaves, W-L-B: winter-low nitrogen-bark, W-L-M: winter-low nitrogen-mature leaves. FC, fold change (winter/spring). ‘Ratio’ is defined as the abundance of a given protein during winter divided by the total protein abundance in the same winter sample. Protein abundance was normalized relative to the overall proteome.

Since N partitioning analysis indicated distinct N remobilization patterns in mature leaves and bark, we further employed proteome analysis to investigate the primary types of storage proteins (mainly 15–35 and 45–55 kDa) in mature leaves and bark (Fig. 5). The VSPs in citrus bark and mature leaves accumulate extensively in winter and decline rapidly in spring, consistent with the seasonal N remobilization pattern. At both high N and low N treatments, we observed the ‘oxygen-evolving enhancer protein 1’ within the 15- to 35-kDa range in the bark. The content of this protein exhibited a substantial increase during winter and decreased during spring (*P* < 0.05, Fig. 5A and B). In the bark, the content of ‘oxygen-evolving enhancer protein 1’ in winter amounted to ca. 0.16%–0.22% of total protein. At high N supply, also the ‘40S ribosomal protein S8’ and ‘60S ribosomal protein L13/L27’ were largely increased in the bark in winter and significantly decreased in spring (*P* < 0.05). At both high N and low N supplies (Fig. 5C and D), the ‘ATP-dependent RNA helicases’, ‘methanethiol oxidase’, and ‘elongation factor’ significantly decreased in spring compared to winter (*P* < 0.05) in the 45- to 55-kDa range of mature leaves. Furthermore, the contents of ‘vacuolar proton pump subunit B’, ‘Elongation Factor 1-alpha’, and ‘ATP-dependent RNA helicases’ reached 0.14%, 0.14%, and 0.10%, respectively, of total protein content in mature leaves at high N supply during winter. At low N supply, the ‘ATP-dependent RNA helicases’ showed the highest content in winter and accounted for as much as 0.11%, of total protein. At low N supply, Rubisco was significantly reduced in mature leaves in spring compared to winter. Overall, ‘oxygen-evolving enhancer protein 1’ is likely to constitute the major VSP in citrus bark, while multiple potential VSPs have been found in mature leaves.

### Key metabolic pathways and molecules in bark and mature leaves

**Figure 6:**
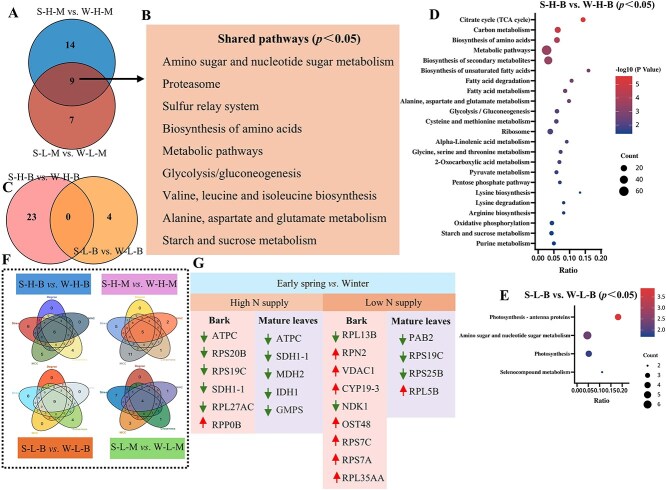
Proteome analysis of KEGG metabolic pathways and hub proteins in mature citrus leaves and bark at varying soil nitrogen (N) contents. (A) The Venn analysis of metabolic pathways enriched in citrus mature leaves under both low N and high N soil conditions. (B) The 9 KEGG pathways (*P* < 0.05) included in A. (C) The Venn analysis of metabolic pathways enriched in citrus bark under both low N and high N soil conditions. (D) The specific KEGG metabolic pathways (S-H-B vs. W-H-B). (E) The specific KEGG metabolic pathway (S-L-B vs. W-L-B). (F) The use of five taxonomic methods and Venn analysis in the four compared groups (S-H-B vs. W-H-B, S-H-M vs. W-H-M, S-L-B vs. W-L-B, and, S-L-M vs. W-L-M) using the Cytoscape and CytoHubba plugins. (G) The specific hub protein names in the four compared groups. Downward-pointing arrows indicate downregulated proteins; upward-pointing arrows indicate upregulated proteins. S-H-B: spring-high nitrogen-bark, S-H-M: spring-high nitrogen-mature leaves, S-L-B: spring-low nitrogen-bark, S-L-M: spring-low nitrogen-mature leaves, W-H-B: winter-high nitrogen-bark, W-H-M: winter-high nitrogen-mature leaves, W-L-B: winter-low nitrogen-bark, W-L-M: winter-low nitrogen-mature leaves.

Apart from storage proteins, we also aimed to identify key pathways reflected by differentially expressed proteins detected through proteome (Fig. 6). At both low and high N supplies, mature leaves exhibited several shared KEGG pathways, i.e. ‘amino sugar and nucleotide sugar metabolism’, ‘proteasome’, ‘sulfur relay system’, ‘biosynthesis of amino acids’, ‘glycolysis/gluconeogenesis’, ‘valine, leucine, and isoleucine biosynthesis’, ‘alanine, aspartate and glutamate metabolism’ and ‘starch and sucrose metabolism’ (Fig. 6A and B). However, no significantly stimulated pathways with common expression were found in the bark at low and high N supplies (Fig. 6C). Compared with high N supply in winter, pathways significantly enriched in the bark at high N supply in spring were mainly related to ‘TCA cycle’, ‘carbon metabolism’, ‘biosynthesis of secondary metabolites’, ‘fatty acid degradation and metabolism’, ‘ribosome’, ‘arginine biosynthesis’, ‘glycolysis/gluconeogenesis’, etc. (Fig. 6D). Compared with low N supply in winter, the pathways significantly enriched in the bark at low N supply in spring were mainly related to ‘photosynthesis-antenna proteins’, ‘amino sugars and nucleotide sugar metabolism’, and ‘photosynthesis’ (Fig. 6E). Overall, in mature leaves and bark, the process of N remobilization involved the pathways of amino acid, sugar, and sulfur metabolism. In addition to these pathways, N remobilization pathway in the bark at high N supply also involved ribosomes, carbon, and fatty acid metabolism. Photosynthesis may be a key pathway at low N supply in both mature leaves and bark.

The proteome results also reveal key molecules that potentially influence N remobilization. Venn analysis was applied to identify hub proteins based on five algorithms (Fig. 6F and G). At high N supply, in bark and mature leaves, energy synthesis proteins (ATPC) and succinate dehydrogenase (SDH1–1) were downregulated in spring compared to winter. In addition, ribosomal proteins (RPS20B, RPS19C, RPL27AC), downregulated exclusively in the bark in spring compared to winter, were found at high N supply. In mature leaves, malate dehydrogenase (MDH2), isocitrate dehydrogenase (IDH1), and guanosine monophosphate synthase (GMPS) were exclusively downregulated under these conditions. At low N supply, one ribosomal protein (RPL13B) was downregulated and four were upregulated (RPN2, RPS7C, RPS7A, RPL35AA) in the bark in spring compared to winter. In addition, mitochondrial outer membrane protein porin (VDAC1), peptidyl-prolyl cis-trans isomerase (CYP19–3), nucleoside diphosphate kinase (NDK1), and protein glycosyltransferase (OST48) were upregulated under these conditions. Compared with low N supply in winter, two ribosomal proteins (RPS19C, RPS25B) and a factor for promoting protein synthesis (PAB2) were downregulated, and one ribosomal protein (RPL5B) was upregulated in mature leaves at low N supply in spring. Apparently, ribosomal proteins constitute key molecules for driving N remobilization in bark and mature leaves.

### Weighted co-expression network analysis of key molecules in mature leaves and bark

**Figure 7:**
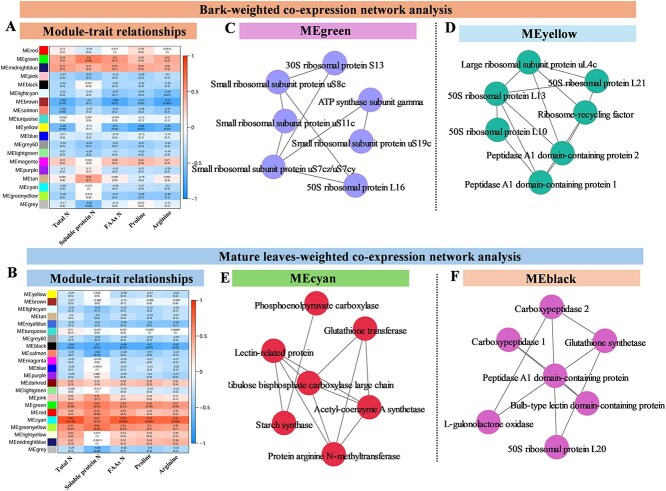
Proteome analysis based on WGCNA. (A and B) The correlation and significance between physiological traits and color modules of mature leaves and bark. The numbers in parentheses indicate significance, while the numbers outside parentheses indicate correlations. (C) The hub proteins of the green module in A. (D) The hub proteins of the yellow module in A. (E) The hub proteins of the cyan module in D. (F) The hub proteins of the green module in D.

To identify key proteins linked to N remobilization, we used weighted co-expression network analysis (WGCNA) to cluster proteins with similar expression patterns into color-coded modules (MEcolor modules), and selected modules strongly correlated with N remobilization traits to pinpoint hub proteins involved (Fig. 7). With this approach, the proteome and N partitioning data were used to find key color modules (Fig. 7A and B). For bark, among all color modules, MEgreen and MEyellow color modules showed positive and negative correlations of proteins with N-partitioning indicators, respectively (Fig. 7A). In the MEgreen color module (Fig. 7C), these proteins included a series of ribosomal proteins, such as 50S/30S ribosomal protein L16/S13, small ribosomal subunit protein us7cz/uS7cy/uS19c/us8c/uS11c, and an ATP synthase subunit gamma. In the MEyellow color module (Fig. 7D), we found a series of ribosomal proteins, including 50S ribosomal protein (L21, L13, L10), a ribosome-recycling factor, large ribosomal subunit protein ul4c as well as a peptidase A1 domain-containing protein.

For mature leaves, among all color modules, MEcyan and MEblack color modules showed positive and negative correlations of proteins with N-partitioning indicators, respectively (Fig. 7B). In MEcyan color modules (Fig. 7E), these proteins included ribulose bisphosphate carboxylase large chain, protein arginine N-methytransferase, and others. In MEbalck color modules (Fig. 7F), we found 50S ribosomal protein L20, peptidase A1 domain-containing protein, and so on. In summary, the WGCNA analysis indicated that the abovementioned proteins may be hub molecules involved in N remobilization.

## Discussion

The present results show that the diversity of N storage in tissues/organs of evergreen citrus trees results in a complexity of physiological mechanism involved in N remobilization as previously suggested for temperate tree species [17]. We revealed that both mature leaves and bark of evergreen citrus trees remobilize stored N for growth during spring. However, distinct remobilization strategies were observed: during spring N mobilization, bark preferentially mobilizes N from stored proteins in the 15- to 35-kDa range, distinct from leaves that stored proteins in the 45- to 55-kDa range. Ribosomal proteins play a key role in N mobilization, with their expression influenced by soil N availability. Under high N supply, bark provides more arginine for N remobilization in early spring, whereas under low N supply, mature leaves respond by supplying more proline in late spring.

### In early spring ,more N resources are remobilized in the bark of citrus trees than in mature leaves

The current results indicate that during early spring, the remobilization of total N and soluble protein N in citrus bark is higher than that in mature leaves, and this phenomenon is more pronounced under high N supply. Apparently, during the N remobilization process in early spring, the stored N resources in bark are preferentially and extensively remobilized compared to mature leaves. This effect may be related to the structure and function of bark and leaves in woody plants [36]. The bark samples collected in this study consisted of the secondary phloem and cortex of the main stems, representing the inner bark tissues actively involved in N storage and remobilization. Although mature leaves are anatomically closer to apical growth points, their N remobilization requires export through petiole vascular bundles to reach the stem’s main transport pathways. In contrast, N stored in the inner bark of main stems can be directly remobilized to support spring shoot growth via the vascular system, reducing dependency on nitrogen translocation from leaves. Being an integral part of the vascular system, the inner bark ensures highly efficient N transport to developing shoots, even compared to spatially closer leaves. Therefore, the transport of N resources from the bark appears to be more convenient. Furthermore, the remobilization process of N requires energy to ensure the loading and unloading of FAAs [37]. Therefore, providing more N resources directly from bark, which is closer to the main stem, seems to reduce energy and material losses. Secondly, bark proteins identified as VSPs primarily function in N storage with minimal catalytic roles, allowing rapid and efficient degradation. In contrast, leaf proteins (e.g. Rubisco) often possess dual functions—both catalytic activity and N storage—meaning their extensive degradation during spring could potentially impact leaf metabolism and photosynthetic efficiency [11–13]. Bark-specific VSPs (e.g. 15–35 kDa proteins) lack enzymatic functions and are thus more ‘economical’ to degrade without compromising tissue functionality. Numerous studies have identified bark as a key N storage organ in woody plants, reinforcing its significance in citrus [7, 38]. Proteins exclusively acting as VSPs remain stable during storage and can rapidly release N resources when needed without affecting metabolic functions [6]. Therefore, from a resource allocation perspective, it appears more suitable to preferentially degrade storage proteins in bark.

In addition, during early spring when temperatures are still relatively low, mature leaves may reduce the export of amino acids with compatible solute functions as a defense against the low temperature in the environment. Proline is a typical compatible solute that can support leaves to withstand low temperatures and is the amino acid with the highest proportion in citrus phloem sap [39]. It is also an important N source for new shoot growth [23]. However, despite its crucial role in supporting leaves under low temperatures, our current results indicate that mature leaves do not significantly remobilize proline during the early spring, and proline is only remobilized in large quantities during the late spring. So, where does the N required for new shoot growth and development come from during the early spring? We found that during early spring, N remobilized in the bark rely on arginine, different from mature leaves. Furthermore, the ‘arginine biosynthesis’ pathway is significantly stimulated under high N supply in the bark during the early spring. Similarly, in other deciduous or temperate trees, arginine has been reported as an important N resource for N storage during winter [3, 10, 17]. With its high N content, arginine is more efficient for the allocation of N resources compared to proline. Furthermore, proline may play a more crucial role during low temperature events in early spring compared to late spring. Under low N supply, proline content in mature leaves decreased significantly in late spring compared to winter. This temporal shift in proline remobilization likely reflects its dual physiological roles: (1) during early spring, proline accumulation in mature leaves may act as a compatible solute to mitigate low temperature-induced cellular damage, delaying its degradation until late spring when temperatures stabilize; (2) under N limitation, proline serves as a critical N reservoir, with its catabolism synchronized to meet the peak demand for new shoot growth in late spring. Therefore, unlike in late spring, neither the bark nor mature leaves significantly remobilize large amounts of proline to new tissues during the early spring stage. In summary, in the bark of citrus trees, more N resources are mobilized during the early spring than in mature leaves to support spring growth (Hypothesis 1).

### Citrus bark and mature leaves contain different VSPs

VSPs are proteins that accumulate in mature tissues as a result of N remobilization from senescing leaves before abscission and are subsequently degraded during active growth phases (e.g. spring) to support new tissue development [3]. SDS-PAGE analyses revealed that VSPs in mature citrus leaves are primarily concentrated within the molecular weight range of 45 to 55 kDa, while those in bark are mainly confined to the range of 15 to 35 kDa. Variations in tree species influence the type of VSPs [3, 13], consistent with our Hypothesis 2. The present study further demonstrates that ‘oxygen-evolving enhancer protein 1 (OEE)’ accumulates at higher levels in citrus bark during winter and decreases significantly in the following spring. The addition of N fertilizer has been reported to significantly enhance the expression of OEE in evergreen trees leaves during winter [19, 20]. The main function of OEE is to participate in the oxygen release during photosynthesis [40]. Photosynthetic proteins and OEE are present in the bark of deciduous *Salix matsudana* [41], and the green color of citrus bark further indicates its involvement in photosynthesis [36, 42]. Our proteomic results reveal that OEE in citrus bark exhibits a seasonal pattern typical of VSPs—it accumulates to high levels during winter, when photosynthetic N demand is lower, and is rapidly degraded in spring. This rapid turnover and high abundance suggest that OEE functions as a temporary N reservoir, releasing stored N to support new growth during seasonal transitions. This suggests that the dual function of OEE may not be unique to citrus but represents a common strategy among evergreens for efficient N management. Moreover, its enhanced expression in photosynthetically active bark cells likely boosts local oxygen evolution, which can help alleviate hypoxia in internal stem tissues where gas diffusion is restricted [43]. VSPs often have dual functions. Thus, OEE can be considered as a potential VSP in citrus bark, also playing a role in photosynthesis while being stored during autumn and winter when light is limited. This mechanism allows the bark to maintain oxygen evolution capacity even when photosynthesis is restricted during these seasons.

Unlike in bark, multiple proteins in the 45- to 55-kDa range, such as ‘vacuolar proton pump subunit B’ and Rubisco, are highly expressed in winter and significantly reduced in spring in mature citrus leaves. Due to the reduced demands for CO₂ fixation in winter, less Rubisco may be needed in leaves during winter. It is still observed in spring that the protein content of Rubisco in the leaves is reduced compared to the winter, which indicates that this protein is also partially used for N storage and mobilization. Given its dominance in leaf soluble protein (exceeding 30%–65% of total leaf protein), with high molecular weight (550 kDa) [44], Rubisco serves as a transient N reservoir that is rapidly degraded in spring to release amino acids for new growth [9]. In addition, VSPs found in the mature leaves of citrus under field conditions have been identified as Rubisco and peptidase A1 domain-containing protein [23]. The results of WGCNA analyses of the present pot experiment also indicate that both Rubisco and peptidase A1 domain-containing protein are important proteins in mature citrus leaves. Apparently, the results of this pot experiment further verify the reliability of the previous field studies [23]. In addition to its key role in carbon fixation, the significant decrease in Rubisco abundance from winter to spring suggests that its N may be reallocated to support new growth. These changes imply that beyond their primary function in photosynthesis, these proteins may also serve as important N reservoirs, contributing to the dynamic process of N remobilization during seasonal transitions.

### Key proteins driving N remobilization in bark and mature leaves of citrus trees

According to Hypothesis 3, both protein–protein interaction (PPI) and WGCNA analyses show that specific ribosomal proteins (RPs) are key regulators of N remobilization in citrus bark and leaves during spring. Similar observations in poplar bark link RP expression to the transition from dormancy to active metabolism [18]. Although RPs primarily function in protein synthesis [45, 46], their N-responsive expression suggests a dual role in N remobilization by influencing VSPs turnover. RP expression in senescing leaves of *Arabidopsis* and maize further supports their involvement in protein degradation and autophagy processes [47, 48]. Under high N supply, a series of RPs were identified in the spring bark, but not in mature leaves in the present study. However, under low N supply, RPs were identified in both spring bark and mature leaves. This observation suggests that under N deficiency, citrus trees need to more actively recycle N stored in mature tissues. At this time, both the bark and mature leaves require RPs to act as central molecules to influence the turnover of storage proteins to recycle N resources, and some RPs may be degraded to even serve as an N source. Under surplus N supply, citrus trees may prioritize the reuse of inactive storage proteins in the bark, as most active proteins in mature leaves are required for catalytic reactions. Proteome results also confirmed that the ‘Ribosome’ pathway was significantly enriched in the bark under high N supply in spring, but not in mature leaves under the same conditions. Exogenous application of N sources (glutamine) inhibited the expression of multiple RPs in poplar leaves [49]. In barley, the expression of RPs increases after the addition of exogenous ammonium, but decreases under long-term N deficiency [50]. These studies indicate that the expression of RPs is influenced by the plant’s N nutritional status as also indicated from the present results. For the key molecules affecting N mobilization in citrus bark and leaves, (1) at high N nutrition, RPs may be key molecules influencing N remobilization in the bark rather than in mature leaves of citrus trees; (2) at N deficiency, RPs may serve as key molecules for N remobilization in both the bark and mature leaves. It is important to emphasize that these RPs function as factors of N remobilization, rather than acting as VSPs. Further research is needed to fully elucidate the multifunctionality of RPs in N remobilization.

## Conclusions

The current results indicate that citrus bark and mature leaves have significantly different N mobilization patterns. Citrus trees remobilize more N resources from the bark than from mature leaves in early spring. In this context, at high N supply, more arginine is mobilized during N remobilization in early spring in the bark, while at low N supply, proline is extensively mobilized in mature leaves in late rather than early spring. In addition, citrus bark contains 15–35 kDa of VSPs, while mature leaves have 45–55 kDa of VSPs. OEE is a bark storage protein, whereas mature leaves store N mainly in proteins like Rubisco and vacuolar proton pump subunit B. Furthermore, proteome analysis identified RPs as key factors of N remobilization in citrus trees during spring. Under high N supply, RPs are crucial molecules for influencing N remobilization in bark, while under low N supply, RPs become key molecules for N remobilization in both bark and mature leaves. We recommend reducing early spring N input by utilizing bark’s priority in N remobilization, especially when low temperatures limit root N uptake, followed by targeted foliar N supplementation in late spring as mature leaves increasingly contribute to N supply with rising temperatures. In future studies, the contribution of citrus roots to N remobilization during spring needs to be investigated.

**Figure 8:**
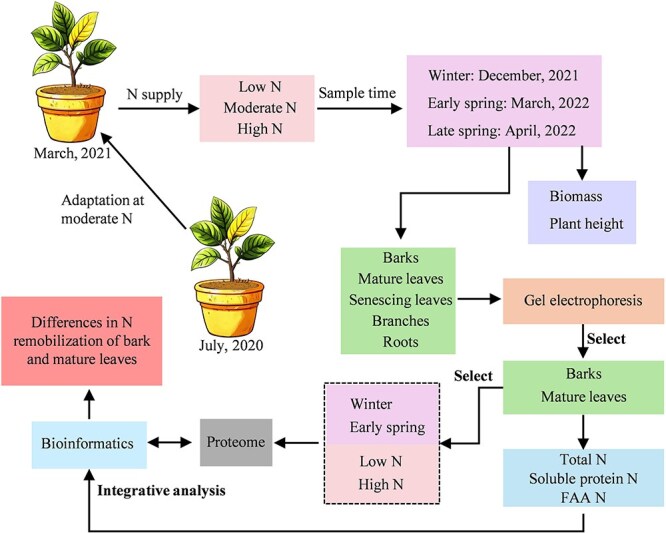
Experimental design of nitrogen (N) fertilizer application of potted citrus seedlings. From July 2020 to March 2021, potted seedlings were acclimated to moderate N, followed by the application of low, moderate, and high N treatments. The experiment spanned three periods: winter, early spring, and late spring. After collecting bark, mature leaves, senescing leaves, branches, and roots across seasons, gel electrophoresis was performed. Based on the results, bark and mature leaves were selected for total N, soluble protein N, and FAA N analysis. Proteome profiling was conducted on bark and mature leaves from winter and early spring under low and high N treatments, followed by integrated analysis of proteome and N partitioning data.

## Materials and methods

### Climatic characteristics of the test area

This field study was conducted in Changshou County, Chongqing City, China (29° 59′ N, 107° 13′E), a humid subtropical climate zone, with four distinct seasons, abundant heat, and precipitation. During the study period from 2020 to 2021, the average annual rainfall was approximately 1600 mm, the average annual temperature about 18°C, and the annual sunshine amounted to 1425 h (https://data.cma.cn). For more detailed local climate information, please refer to previous studies [51].

### Experimental design and fertilization management

The citrus trees used in this experiment were 2-year-old seedlings with ‘Ziyang’ (*Citrus × junos Siebold ex Tanaka*) rootstock and ‘Dayagan’ scion (*Citrus reticulata* Blanco). This hybrid is widely planted by farmers in Southwest China [52]. Citrus seedlings were transplanted in July 2020 into pots (upper: 37.5 cm, lower: 30 cm, height: 40 cm) that were equipped with water outlet holes at the bottom, and they were cultivated until March 2021. Each pot was filled with 35 kg of dry soil; the soil type was an entisol according to the United States Department of Agriculture soil taxonomy [53]. The basic nutrient composition of the soil before the experiment was as follows: pH 5.04; organic matter, 12.7 g·kg^−1^; total N, 0.5 g·kg^−1^; available phosphorus, 50.50 g·kg^−1^; and available potassium, 115 g·kg^−1^. In March 2021, visually consistent and healthy citrus seedlings were selected, and three N level treatments (low N, moderate N, and high N) were applied. Each N level treatment had three replications, and each replication had four potted citrus plants, for a total of 36 potted citrus seedlings. Based on the age of the potted citrus trees and previous studies, the annual N dosages of the low N treatment, the moderate N treatment, and the high N treatment were 2.10, 8.40, and 16.81 g N/plant, respectively [33, 54]. The annual N fertilizer use was divided into eight equal parts applied every month from March to October 2021. Phosphorus and potassium were applied in one step in March 2021 at each N level as calcium superphosphate and potassium sulfate at 2.63 g P_2_O_5_/plant and 5.25 g K_2_O per plant, respectively [33], consistent with local fruit farmers’ fertilizer management. In 2022, the N application was repeated in March 2022 at 80% of the annual application rate applied in 2021 for all N levels. At the same time, superphosphate and potassium sulfate were applied at the same dose as in 2021. All potted citrus trees were exposed to under natural fluctuating environmental conditions outdoors (Fig. 8).

### Sample collection and processing

Destructive sampling of citrus trees was performed in December 2021 and March 2022. Before dissecting, the tree height was measured, and the trees were immediately weighed to obtain the total fresh biomass. Additionally, to perform gel electrophoresis in different tissues (see specific methods below), fresh samples of mature leaves (defined as fully expanded, healthy leaves that began growing in autumn 2021 and were primarily collected from the mid-canopy to ensure photosynthetic activity), senescing leaves (identified by visible yellowing and typically collected from the lower canopy), as well as inner bark [7], branches, and roots were collected in winter (December 2021), early spring (March 2022), and late spring (April 2022). Specifically, the bark samples were collected by gently scraping the trunk surface with a paper knife, and the outer bark (dead tissue) was carefully removed to obtain the living inner bark (secondary phloem and cortex). A section of the root system was dug out, and roots were cut from the taproot. The samples collected were stored at −80°C for subsequent analyses. A portion of fresh mature leaf and bark samples were oven-dried in an oven at 65°C for 72 h until weight constancy to determine the water content.

### Determination of physiological indicators

The total N contents of mature leaves and bark were measured using the Kjeldahl method [55]. In brief, after samples were digested by sulfuric acid and dried, sodium hydroxide was added followed by distillation. Finally, ammonia was absorbed using a boric acid indicator. For the extraction of soluble protein, 0.05 g of tissue was homogenized in 1.0 ml of distilled water in an ice bath. The mixture was centrifuged at 8000*g* and 4°C for 10 min. The resulting supernatant was collected and kept on ice for subsequent analysis. The soluble protein content in leaves and bark was quantified using the Coomassie brilliant blue method with bovine serum albumin as standard [56].

Quantification of 17 FAAs in citrus leaves and bark was achieved using high-performance liquid chromatography (Ultimate 3000, Thermo Fisher Scientific Inc., Germany) with a Kromasil C18 column and a ultraviolet–visible detector (UV–V is detector) as previously described [21]. Briefly, the mobile phase A contained 7.6 g of anhydrous sodium acetate dissolved in 925 ml of distilled water adjusted to pH 6.5 with glacial acetic acid. Subsequently, 70 ml of acetonitrile was added, mixed evenly, and filtered through a 0.45-μm filter. Mobile phase B consisted of 80% acetonitrile in distilled water. For amino acid extraction, 0.10 g of tissue was added to 1.0 ml of ultrapure water, grinded to a slurry, and transferred to an Eppendorf tube (Hamburg, Germany) for overnight extraction, followed by filtration through a 0.22-μm filter membrane. An aliquot of 200 μl of supernatant and 200 μl of amino acid standard solution (Sigma–Aldrich, MO, USA) was placed in 1.5-ml Eppendorf tubes, and 20 μl of L-norvaline internal standard solution was added. Subsequently, 200 μl of triethylamine acetonitrile (pH > 7) and 100 μl of isothiocyanatobenzene acetonitrile were added and incubated at 25°C for 1 h. Finally, 400 μl of n-hexane were added for 10 min, with the lower solution layer collected and filtered using a 0.45-μm syringe filter. The concentration of each FAA was quantified using a custom-made calibration curve of the external standard solution (Sigma-Aldrich, Missouri, USA).

### Proteome analysis

The procedure of proteome analysis included protein extraction, sodium dodecyl sulfate polyacrylamide gel electrophoresis (SDS-PAGE), trypsin hydrolysis, liquid chromatography–tandem mass spectrometry (LC–MS/MS) detection, and database search [23]. Briefly, samples were ground under liquid N_2_, an extraction solution (150 mM Tris HCl [pH 8.0], 2% polyvinylpolypyrrolidone, 25% glycerol) was added, and samples were centrifuged at low temperature and high speed. An aliquot of 200 μl of the collected supernatant was added to 200 μl of 8 mol/L urea solution and 200 μl of ultrapure water. The mixture was subjected to low-temperature high-speed centrifugation and the supernatant used for SDS-PAGE. For this purpose, an appropriate amount of sample was mixed with loading buffer and heated in boiling water for 5 min. Then, the samples and markers were added to the wells of a 12% resolving gel for 75 min, stained with Coomassie brilliant blue staining solution for 1 h, and subsequently exposed to a destaining solution. For trypsin digestion, micellar decolorization (50% ACN–50% 50 mmol/L NH_4_HCO_3_), micellar dehydration (100% ACN, 500 μl), reductive alkylation (50% ACN–50% NH_4_HCO_3_, 50% ACN–50% 50 mmol/L , 10 mmol/L DTT, 55 mmol/L iodoacetamide, 100% ACN), enzyme digestion (15 ng/μl trypsin and 50 mmol/L NH_4_HCO_3_), peptide extraction (5% TFA–50% ACN–45% water), desalting (self-priming desalting column, Thermo Fisher Scientific, New York, USA), and vortexing (0.1% formic acid, 2% acetonitrile) were performed. For subsequent LC–MS/MS analysis (Easy-nLC 1200, Thermo Fisher Scientific, Waltham, MA, USA) of the resulting peptides, a nanocolumn (150 μm × 15 cm) in-house packed with Acclaim PepMap RPLC C18 resin (1.9 μm, 100 Å, Dr. Maisch GmbH, Germany) was applied. Gradient elution was performed with mobile phase A (0.1% formic acid aqueous solution) and mobile phase B (0.1% formic acid aqueous solution–80% acetonitrile).

### Profiling analysis of the proteome

A total of four comparison groups were set up in this experiment, namely, ‘spring vs. winter’ under time series analysis (including ‘S-H-B vs. W-H-B’, ‘S-H-M vs. W-H-M’, ‘S-L-B vs. W-L-B’, and ‘S-L-M vs. W-L-M’). S represents early spring, W winter, L low soil N, H high soil N, M mature leaves, and B bark. Proteins identified in citrus trees were named and functionally identified in the UniProt database (https://www.uniprot.org/). Differentially expressed proteins were subjected to homology comparison with *Arabidopsis*. The identified proteins were then mapped to the KEGG (Kyoto Encyclopedia of Genes and Genomes) database to visualize metabolic pathways. A PPI network was created using the online website STRING (http://www.string-db.org/) and the Cytoscape software (version 2.8.3, San Diego, CA, USA) [57]. Based on the PPI results, potential key proteins were identified by utilizing the CytoHubba plugin within the Cytoscape software (v3.10.1, San Diego, CA, USA) and setting up a Venn diagram with five taxonomic (degree, maximum neighborhood component [MNC], stress, closeness and radiality) methods [58, 59].

### Weighted co-expression network analysis

Proteome-wide analysis was conducted using the WGCNA package in R [60]. The parameters were set as follows: soft power 2, minModuleSize 50, deepSplit 2, and mergeCutHeight 0.25. The regulation of module-associated pathways is often influenced by core (hub) proteins within the network. To identify the core proteins that play important regulatory roles in this module, the protein regulatory network was analyzed using Cytoscape software (v3.10.1, San Diego, CA, USA) framework with visualization processing [58].

### Statistic analyses

Nitrogen partitioning, SDS-PAGE, and label-free proteome analysis were performed with three replicate groups (*n* = 3) for each treatment. Nitrogen partitioning data were subjected to normality tests (Shapiro–Wilk method) and successfully passed, indicating that all data were normally distributed. Subsequently, two-way significance tests (Tukey’s test, *P* < 0.05) were performed using the GraphPad Prism 8.0 software (San Diego, CA, USA). Histograms were created using GraphPad Prism. Pathway enrichment maps, Venn diagrams, network diagrams, and heatmaps were created with online programs (https://www.omicshare.com/tools, https://hiplot.com.cn).

## Data Availability

The MS proteomics data have been deposited to the ProteomeXchange Consortium (http://proteomecentral.proteomexchange.org) via the iProX partner repository with the dataset identifier PXD055529. The corresponding author of this study can provide raw data based on reasonable grounds.

## References

[1] Chen K-E, Chen H-Y, Tseng C-S. et al. Improving nitrogen use efficiency by manipulating nitrate remobilization in plants. Nat Plants. 2020;6:1126–35 32868892 10.1038/s41477-020-00758-0

[2] Cao J, Zheng X, Xie D. et al. Autophagic pathway contributes to low-nitrogen tolerance by optimizing nitrogen uptake and utilization in tomato. Hortic Res. 2022;9:uhac068 35669705 10.1093/hr/uhac068 PMC9164271

[3] Millard P, Grelet G-A. Nitrogen storage and remobilization by trees: ecophysiological relevance in a changing world. Tree Physiol. 2010;30:1083–95 20551251 10.1093/treephys/tpq042

[4] Tegeder M, Masclaux-Daubresse C. Source and sink mechanisms of nitrogen transport and use. New Phytol. 2018;217:35–53 29120059 10.1111/nph.14876

[5] Abbaraju HKR, Gupta R, Appenzeller LM. et al. A vegetative storage protein improves drought tolerance in maize. Plant Biotechnol J. 2022;20:374–89 34614273 10.1111/pbi.13720 PMC8753363

[6] Rennenberg H, Wildhagen H, Ehlting B. Nitrogen nutrition of poplar trees. Plant Biol. 2010;12:275–91 20398235 10.1111/j.1438-8677.2009.00309.x

[7] Rosell JA, Marcati CR, Olson ME. et al. Inner bark vs sapwood is the main driver of nitrogen and phosphorus allocation in stems and roots across three tropical woody plant communities. New Phytol. 2023;239:1665–78 37381089 10.1111/nph.19085

[8] Langheinrich U, Tischner R. Vegetative storage proteins in poplar: induction and characterization of a 32-and a 36-kilodalton polypeptide. Plant Physiol. 1991;97:1017–25 16668485 10.1104/pp.97.3.1017 PMC1081118

[9] Cooke JE, Weih M. Nitrogen storage and seasonal nitrogen cycling in populus: bridging molecular physiology and ecophysiology. New Phytol. 2005;167:19–30 15948826 10.1111/j.1469-8137.2005.01451.x

[10] Wildhagen H, Dürr J, Ehlting B. et al. Seasonal nitrogen cycling in the bark of field-grown grey poplar is correlated with meteorological factors and gene expression of bark storage proteins. Tree Physiol. 2010;30:1096–110 20354193 10.1093/treephys/tpq018

[11] Wyka TP, Żytkowiak R, Oleksyn J. Seasonal dynamics of nitrogen level and gas exchange in different cohorts of scots pine needles: a conflict between nitrogen mobilization and photosynthesis? Eur J For Res. 2016;135:483–93

[12] Proe MF, Midwood AJ, Craig J. Use of stable isotopes to quantify nitrogen, potassium and magnesium dynamics in young scots pine (pinus sylvestris). New Phytol. 2000;146:461–9

[13] Tian W-M, Peng S-Q, Wang X-C. et al. Vegetative storage protein in litchi chinensis, a subtropical evergreen fruit tree, possesses trypsin inhibitor activity. Ann Bot. 2007;100:1199–208 17913726 10.1093/aob/mcm216 PMC2759257

[14] Feller U, Anders I, Mae T. Rubiscolytics: fate of rubisco after its enzymatic function in a cell is terminated. J Exp Bot. 2008;59:1615–24 17975207 10.1093/jxb/erm242

[15] Seidel F, Lopez CML, Oikawa A. et al. Seasonal nitrogen partitioning in Japanese cedar (*Cryptomeria japonica*, D. Don) tissues. Plant and Soil. 2019;442:511–29

[16] Warren CR, Adams MA. Evergreen trees do not maximize instantaneous photosynthesis. Trends Plant Sci. 2004;9:270–4 15165557 10.1016/j.tplants.2004.04.004

[17] Babst BA, Coleman GD. Seasonal nitrogen cycling in temperate trees: transport and regulatory mechanisms are key missing links. Plant Sci. 2018;270:268–77 29576080 10.1016/j.plantsci.2018.02.021

[18] Islam N, Li G, Garrett WM. et al. Proteomics of nitrogen remobilization in poplar bark. J Proteome Res. 2015;14:1112–26 25513840 10.1021/pr501090p

[19] Zhang S, Zhang L, Chai Y. et al. Physiology and proteomics research on the leaves of ancient *Platycladus orientalis* (L.) during winter. J Proteomics. 2015;126:263–78 26159399 10.1016/j.jprot.2015.06.019

[20] Zhang Y, Han Q, Guo Q. et al. Physiological and proteomic analysis reveals the different responses of *Cunninghamia lanceolata* seedlings to nitrogen and phosphorus additions. J Proteomics. 2016;146:109–21 27389851 10.1016/j.jprot.2016.07.001

[21] Xiong H, Ma H, Zhao H. et al. Integrated physiological, proteome and gene expression analyses provide new insights into nitrogen remobilization in citrus trees. Tree Physiol. 2022;42:1628–45 35225347 10.1093/treephys/tpac024

[22] Moreno J, García-Martinez JL. Nitrogen accumulation and mobilization in citrus leaves throughout the annual cycle. Physiol Plant. 1984;61:429–34

[23] Xiong H, Luo Y, Zhao H. et al. Integrated proteome and physiological traits reveal interactive mechanisms of new leaf growth and storage protein degradation with mature leaves of evergreen citrus trees. Tree Physiol. 2024;44:tpae001 38195893 10.1093/treephys/tpae001

[24] Laino P, Russo MP, Guardo M. et al. Rootstock–scion interaction affecting citrus response to CTV infection: a proteomic view. Physiol Plant. 2016;156:444–67 26459956 10.1111/ppl.12395

[25] Pagliaccia D, Shi J, Pang Z. et al. A pathogen secreted protein as a detection marker for citrus huanglongbing. Front Microbiol. 2017;8:2041 29403441 10.3389/fmicb.2017.02041 PMC5776943

[26] Roccuzzo G, Zanotelli D, Allegra M. et al. Assessing nutrient uptake by field-grown orange trees. Eur J Agron. 2012;41:73–80

[27] Zhao Y, Xiong H, Luo Y. et al. Long-term nitrogen fertilization alters the partitioning of amino acids between citrus leaves and fruits. Front Plant Sci. 2025;15:1516000 39872200 10.3389/fpls.2024.1516000 PMC11769974

[28] Jones JM, Heineman KD, Dalling JW. Soil and species effects on bark nutrient storage in a premontane tropical forest. Plant and Soil. 2019;438:347–60

[29] Yu S, Zhu M, Li P. et al. Dissection of the spatial dynamics of biosynthesis, transport, and turnover of major amino acids in tea plants (*Camellia sinensis*). Hortic Res. 2024;11:uhae060 38716228 10.1093/hr/uhae060 PMC11070726

[30] Zhao H, Lakshmanan P, Wang X. et al. Global reactive nitrogen loss in orchard systems: a review. Sci Total Environ. 2022;821:153462 35093357 10.1016/j.scitotenv.2022.153462

[31] Liao L, Dong T, Qiu X. et al. Nitrogen nutrition is a key modulator of the sugar and organic acid content in citrus fruit. PLoS One. 2019;14:e0223356 31600253 10.1371/journal.pone.0223356 PMC6786551

[32] Roccuzzo G, Scandellari F, Allegra M. et al. Seasonal dynamics of root uptake and spring remobilisation of nitrogen in field grown orange trees. Sci Hortic. 2017;226:223–30

[33] Dovis VL, Erismann NM, Machado EC. et al. Biomass partitioning and photosynthesis in the quest for nitrogen-use efficiency for citrus tree species. Tree Physiol. 2021;41:163–76 33032323 10.1093/treephys/tpaa126

[34] Peng M-Y, Ren QQ, Lai YH. et al. Integration of physiology, metabolome and transcriptome for understanding of the adaptive strategies to long-term nitrogen deficiency in *Citrus sinensis* leaves. Sci Hortic. 2023;317:112079

[35] Chen H, Hu W, Wang Y. et al. Declined photosynthetic nitrogen use efficiency under ammonium nutrition is related to photosynthetic electron transport chain disruption in citrus plants. Sci Hortic. 2023;308:111594

[36] Rosell JA . Bark in woody plants: understanding the diversity of a multifunctional structure. Integr Comp Biol. 2019;59:535–47 31120526 10.1093/icb/icz057

[37] Lalonde S, Tegeder M, Throne-Holst M. et al. Phloem loading and unloading of sugars and amino acids. Plant Cell Environ. 2003;26:37–56

[38] Gong H, Niu Y, Niklas KJ. et al. Nitrogen and phosphorus allocation in bark across diverse tree species. Sci Total Environ. 2024;908:168327 37926252 10.1016/j.scitotenv.2023.168327

[39] Hijaz F, Killiny N. Collection and chemical composition of phloem sap from *Citrus sinensis* L. Osbeck (sweet orange). PLoS One. 2014;9:e101830 25014027 10.1371/journal.pone.0101830 PMC4094394

[40] Mayfield SP, Rahire M, Frank G. et al. Expression of the nuclear gene encoding oxygen-evolving enhancer protein 2 is required for high levels of photosynthetic oxygen evolution in *Chlamydomonas reinhardtii*. Proc Natl Acad Sci. 1987;84:749–53 3468511 10.1073/pnas.84.3.749 PMC304293

[41] Liu J, Sun C, Zhai FF. et al. Proteomic insights into the photosynthetic divergence between bark and leaf chloroplasts in *Salix matsudana*. Tree Physiol. 2021;41:2142–52 33987679 10.1093/treephys/tpab055

[42] Cernusak LA, Hutley LB. Stable isotopes reveal the contribution of corticular photosynthesis to growth in branches of *Eucalyptus miniata*. Plant Physiol. 2011;155:515–23 21078864 10.1104/pp.110.163337 PMC3075781

[43] Wittmann C, Pfanz H. More than just CO 2-recycling: corticular photosynthesis as a mechanism to reduce the risk of an energy crisis induced by low oxygen. New Phytol. 2018;219:551–64 29767842 10.1111/nph.15198

[44] Nawaz MA, Kasote DM, Ullah N. et al. RuBisCO: a sustainable protein ingredient for plant-based foods. Front Sustain Food Syst. 2024;8:1389309

[45] Couso I, Pérez-Pérez ME, Martínez-Force E. et al. Autophagic flux is required for the synthesis of triacylglycerols and ribosomal protein turnover in *Chlamydomonas*. J Exp Bot. 2018;69:1355–67 29053817 10.1093/jxb/erx372 PMC6018900

[46] Byrne ME . A role for the ribosome in development. Trends Plant Sci. 2009;14:512–9 19716746 10.1016/j.tplants.2009.06.009

[47] Li F, Chung T, Pennington JG. et al. Autophagic recycling plays a central role in maize nitrogen remobilization. Plant Cell. 2015;27:1389–408 25944100 10.1105/tpc.15.00158 PMC4456646

[48] Guiboileau A, Avila-Ospina L, Yoshimoto K. et al. Physiological and metabolic consequences of autophagy deficiency for the management of nitrogen and protein resources in *Arabidopsis* leaves depending on nitrate availability. New Phytol. 2013;199:683–94 23647084 10.1111/nph.12307

[49] Han M, Xu M, Su T. et al. Transcriptome analysis reveals critical genes and pathways in carbon metabolism and ribosome biogenesis in poplar fertilized with glutamine. Int J Mol Sci. 2022;23:9998 36077396 10.3390/ijms23179998 PMC9456319

[50] Møller AL, Pedas PAI, Andersen B. et al. Responses of barley root and shoot proteomes to long-term nitrogen deficiency, short-term nitrogen starvation and ammonium. Plant Cell Environ. 2011;34:2024–37 21736591 10.1111/j.1365-3040.2011.02396.x

[51] Luo Y, Xiong H, Zhao H. et al. Accumulated temperature rather than nitrogen fertilization is the main factor determining growth of young citrus trees in the field. Sci Hortic. 2024;323:112511

[52] Hu Z, Wang F, Yu H. et al. Effects of scion-rootstock interaction on citrus fruit quality related to differentially expressed small RNAs. Sci Hortic. 2022;298:110974

[53] Service NRC . Soil Taxonomy: A Basic System of Soil Classification for Making and Interpreting Soil Surveys. Washington, DC: US Department of Agriculture; 1999:

[54] Martínez-Alcántara B, Quiñones A, Primo-Millo E. et al. Nitrogen remobilization response to current supply in young citrus trees. Plant and Soil. 2011;342:433–43

[55] Xu X, Zhang X, Ni W. et al. Nitrogen–potassium balance improves leaf photosynthetic capacity by regulating leaf nitrogen allocation in apple. Hortic Res. 2024;11:uhad253 38486813 10.1093/hr/uhad253 PMC10939330

[56] Wang Y, Song S, Hao Y. et al. Role of *BraRGL1* in regulation of *Brassica rapa* bolting and flowering. Hortic Res. 2023;10:uhad119 37547730 10.1093/hr/uhad119 PMC10402658

[57] Shannon P, Markiel A, Ozier O. et al. Cytoscape: a software environment for integrated models of biomolecular interaction networks. Genome Res. 2003;13:2498–504 14597658 10.1101/gr.1239303 PMC403769

[58] Chin C-H, Chen S-H, Wu H-H. et al. cytoHubba: identifying hub objects and sub-networks from complex interactome. BMC Syst Biol. 2014;8:1–7 25521941 10.1186/1752-0509-8-S4-S11 PMC4290687

[59] Ahmad S, Lu C, Gao J. et al. Integrated proteomic, transcriptomic, and metabolomic profiling reveals that the gibberellin–abscisic acid hub runs flower development in the Chinese orchid *Cymbidium sinense*. Hortic Res. 2024;11:uhae073 38738212 10.1093/hr/uhae073 PMC11088716

[60] Langfelder P, Horvath S. WGCNA: an R package for weighted correlation network analysis. BMC Bioinform. 2008;9:1–13 10.1186/1471-2105-9-559 PMC2631488 19114008

